# Relevance of computerized tomography in the preoperative evaluation of patients with vulvar cancer: a prospective study

**DOI:** 10.1186/s40644-015-0044-2

**Published:** 2015-06-10

**Authors:** Kjeld Andersen, Vibeke Zobbe, Ingrid Regitze Thranov, Karen Damgaard Pedersen

**Affiliations:** Department of Radiology, Rigshospitalet, University Hospital Copenhagen, Blegdamsvej 9, 2100 Copenhagen, Denmark; Department of Obstetrics and Gynecology, Rigshospitalet, University Hospital Copenhagen, Blegdamsvej 9, 2100 Copenhagen, Denmark

**Keywords:** Computerized tomography, Staging, Vulvar cancer, Comorbidity, Logistics, Sentinel node, Incidental findings

## Abstract

**Background:**

The purpose of the study was to determine whether inclusion of computerized tomography (CT) in the prospective evaluation of vulvar cancer changed the surgical treatment strategy in terms of detection of lymph node metastases, tumor spread and comorbidity, and additionally to examine the logistical influence of adding further examinations prior to treating out-hospital patients referred from geographically distant areas.

**Methods:**

During an 8 month period we conducted a prospective study of patients with newly diagnosed or recurrent vulvar cancer consecutively referred to Copenhagen University Hospital, Rigshospitalet. The patients underwent a gynecological examination, chest x-ray and a preoperative CT scanning of the chest, abdomen and pelvis. It was registered whether the radiological findings regarding the extent of the tumor, lymph node involvement, incidental findings and comorbidity changed the surgical treatment plan. Further, the logistical influence of the long referral distances was registered.

**Results:**

Thirty patients with a median age of 69 years (range 44–93 years) were included in the study. CT did not significantly change the initial surgical treatment plan for the patients. CT did not reveal lymph node enlargement outside the inguinofemoral area and was inaccurate compared to the sentinal node examination of the local lymph nodes. CT diagnosed no cases with distant metastases from the primary malignancy, but two cases with a secondary malignant disease were found.

**Conclusions:**

CT scanning has no clinical impact as a routine screening examination prior to surgery. It may delay treatment, but can add important information when clinically indicated.

## Background

Vulvar cancer is a rare malignancy of the external genitals in adult women. The incidence of the disease in Denmark is 80–100 annually and the patients are generally elderly women, with a median age of 65 years [1]. Due to the advanced age of the patients, the prevalence of comorbidity is relatively high [2, 3]. In accordance with the treatment guidelines of the ongoing Groningen International Study on Sentinel Nodes in Vulvar Cancer II (GROINSS-V II) [4], the primary treatment of clinically localized vulvar cancer consists of partial or total vulvectomy. Spread to the inguinal lymph nodes is evaluated either by the sentinel node procedure (vulvar tumor ≤4 cm and no clinical signs of tumor spread to inguinal nodes) or lymphadenectomy of the regional lymph nodes. Danish guidelines used for treatment planning include preoperative gynecological examination and imaging techniques, such as chest x-ray, abdominal or transvaginal ultrasound (UL), and in individual cases either computerized tomography (CT), positron emission tomography (PET) or magnetic resonance imaging (MRI) [5]. Outside Denmark, these supplementary cross-sectional imaging modalities are recommended routinely [6, 7]. However, the literature is scarce regarding the benefit of these preoperative examinations for the decisive surgical procedure and the final outcome of the patients, including identification of lymph node metastases and probable comorbidity and incidental findings detected [8–11]. The final stage of the disease is determined by the combined gynecological, pathological and radiological findings, in accordance to the revised guidelines proposed by the International Federation of Gynecology and Obstetrics (FIGO) [12, 13]. Of special importance is, whether identified comorbidity and incidental findings will moderate or change the scheduled surgical treatment plan or follow-up[14].

In Denmark, treatment has been centralized to two departments, and thus the uptake of patients with vulvar cancer covers a large geographical area. The Gynecological Department, receives patients from the eastern and southern part of Denmark, resulting in long travel distances for a significant number of the patients. The logistics of additional examinations to the existing scheduled procedures might be expensive, time consuming and eventually delaying surgery.

The primary goal of the study was to investigate the clinical impact of adding a preoperative CT scan in the evaluation of invasiveness of the tumor, metastatic spread or incidental abnormalities that might influence the scheduled surgical plan.

## Methods

We performed a prospective study of women consecutively referred to the Gynecological Department at Copenhagen University Hospital, Rigshospitalet, between July 2012 and February 2013. All patients were newly diagnosed with histologically confirmed vulvar carcinoma or recurrence of a vulvar cancer, previously treated and considered cured. The patients were clinically examined by the same two gynecologists (IT and VZ), and the surgical plan was decided according to the Danish guidelines. For all patients this was noted in a prospective registration form. The patient information and strategy described in the registration form followed the patient to the radiologists, who performed a CT scan of the chest, abdomen and pelvis. Concomitantly, chest x-ray examination was performed as a part of the usual preoperative workup. The equipment for CT scan included in all cases a 64-slice multidetector CT scanner (MDCT), and all patients received intravenous and orally administered contrast agent. It was a prerequisite that the serum creatinine was known. All scans were supervised by the same radiologists (KA and KDP) and analyzed within two days. The CT diagnoses included the extension of the primary tumor in the vulvar region, tumor size, degree of invasiveness and especially the relationship to the urethra. The lymph node size, appearance and contrast enhancement was analyzed in the superficial inguinofemoral area, in the minor pelvis and in the retroperitoneal space. On CT scan a lymph node was regarded as malignant if the minimal short axis diameter was exceeding 10 mm in the axial plane, and/or if it presented with an abnormal pattern of contrast enhancement. The analysis further included signs of metastases in the lungs, mediastinum, abdominal organs or bones. Abnormal findings were described in the patient registration form. Finally, the preoperative clinical findings and plan were correlated to the CT findings at an internal conference prior to surgical treatment of the patient. It was noted whether the CT scan changed the initial surgical treatment strategy, including uni- or bilateral sentinel node examination–or in certain cases lymphadenectomy. Further, whether the CT scan visualized important incidental findings influencing the final treatment plan, and finally whether adding a CT scan would delay surgery for the out-hospital patients. The study is in compliance with the Helsinki Declaration. The patients were treated according to the Danish Guidelines for treatment of vulva cancer, which request a CT-, MR- or PET-CT scan preoperatively and the results of the CT scans were revealed to the surgeons pre-operatively. All patients gave written consent to participate in the study and for the results to be published.

## Results

Thirty consecutive patients with newly diagnosed or recurrent vulvar cancer were referred to the gynecological department during the eight month period. Twenty-seven patients were included in the study and all data are listed in Table 1. Three patients were excluded due to lack of a registration form (one patient) or intolerance of the intravenous contrast agent (two patients). The median age of the patients was 69 years (range 44–93 years). A small majority (56 %) of the patients were referred from outside the Copenhagen area (southern and eastern part of Denmark). With some exceptions, all patients were scheduled for a CT scan on the same day or the day after the initial examination (weekends and holidays not included). Chest x-ray was performed with a median of 10 days (range 0–21 days) after the initial examination, and surgery was performed with a median of 12 days later (range 0–22 days). Twenty-three of the patients had newly diagnosed vulvar carcinoma and four had recurrence of the disease. According to the gynecological examination, the tumor size was below 4 cm in 23 cases, and exceeding 4 cm in the remaining four cases. Twenty-one patients were registered as suffering from various forms of comorbidity, e.g. hypertension, hyperthyroidism and diabetes. The initial clinical stage and final stage according to the FIGO classification is listed in Table 2. A total of five patients were upstaged following surgery, lymph node examination and histopathology due to metastatic spread to the inguinal nodes, thus the number of patients with stage I/II was reduced from 24 to 19. However, the findings did not influence the initial surgical treatment strategy. Imaging included chest x-ray and MDCT. Chest x-ray was performed in 24 of the 27 patients. The three exceptions were due to lack of attendance for unknown reasons. Thirteen examinations were normal, ten demonstrated findings of minor clinical importance, and in one case chest x-ray unexpectedly revealed pulmonary nodules, later shown to represent metastases from an asymptomatic unknown adenocarcinoma of the cecum, visualized by CT. Chest x-ray did not change the initial gynecological treatment strategy for any of the patients.

**Table 1:** Characteristics of 27 women with vulvar cancer

Clinical characteristics (*n* = 27)	Median	IQR
Age (years)	69^a^	22^b^
Time from initial examination to intervention		
CT (days)	6^a^	5^b^
Chest x-ray (days)	10^a^	7^b^
Surgery (days)	12^a^	8^b^
Catchment area	n	%
Regional	12	44 %
Extra-regional^c^	15	56 %
Initial gynecological examination		
Recurrence	4	15 %
Debut	23	85 %
Clinically registered comorbidity	21	78 %
Tumor size >4 cm	4	15 %
Incidental findings on CT		
No incidental findings	1	4 %
Minor and moderate importance^d^	21	78 %
Major importance^e^	3	11 %
Confirmed cancers^f^	2	7 %
Histology		
Squamous cell carcinoma	26	96 %
Basosquamous carcinoma	1	4 %

^a,b^ Data are summarized as median values and interquartile ranges (IQR)

^c^Southern and eastern part of Denmark

^d^One or more findings of minor and moderate importance, i.g. atherosclerosis, cholecystolithiasis, simple renal- and hepatic cysts, diverticula and hiatus hernia

^e^Findings of major importance were pulmonary nodules, an endometrial polyp and biliary ectasia

^f^Renal cell carcinoma and adenocarcinoma of the cecum with pulmonary metastases

**Table 2:** Pre- and final postoperative stage of 27 women with vulvar cancer

Preoperative and final postoperative stage (*n* = 27)	Number	Percent
Initial FIGO stage		
IA	1	4 %
IB	20	74 %
II	3	11 %
III	3	11 %
IV	0	0 %
Final FIGO stage		
IA	1	4 %
IB	17	63 %
II	1	4 %
III	7	26 %
IV	1	4 %

CT scan of the chest, abdomen and pelvis was performed on all 27 patients (Table 1). Local invasiveness regarding involvement of the urethra was indicated in three patients. No distant metastases originating from the primary vulvar tumor were found. In seven patients a total of eight ipsilateral lymph nodes with enlargement/hyperdensity after intravenous contrast were found in the groin on the CT scanning. However, the ability of the observer to correctly identify groin lymph node metastases on a CT scan compared to histological examination was poor, with a sensitivity of 60 %, specificity of 90 %, positive predictive value of 37.5 % and negative predictive value of 95.7 % (Table 3).

**Table 3:** Number of groin dissections (*n*). Histological examination of the specimen determined true nodal status

Accuracy of CT in detecting lymph node metastases in the groin		
	Abnormal on histological examination (*n* = 10)	Normal on histological examination (*n* = 44)
Abnormal on CT (*n* = 8)	3	5
Normal on CT (*n* = 46)	2	44

**Fig. 1:**
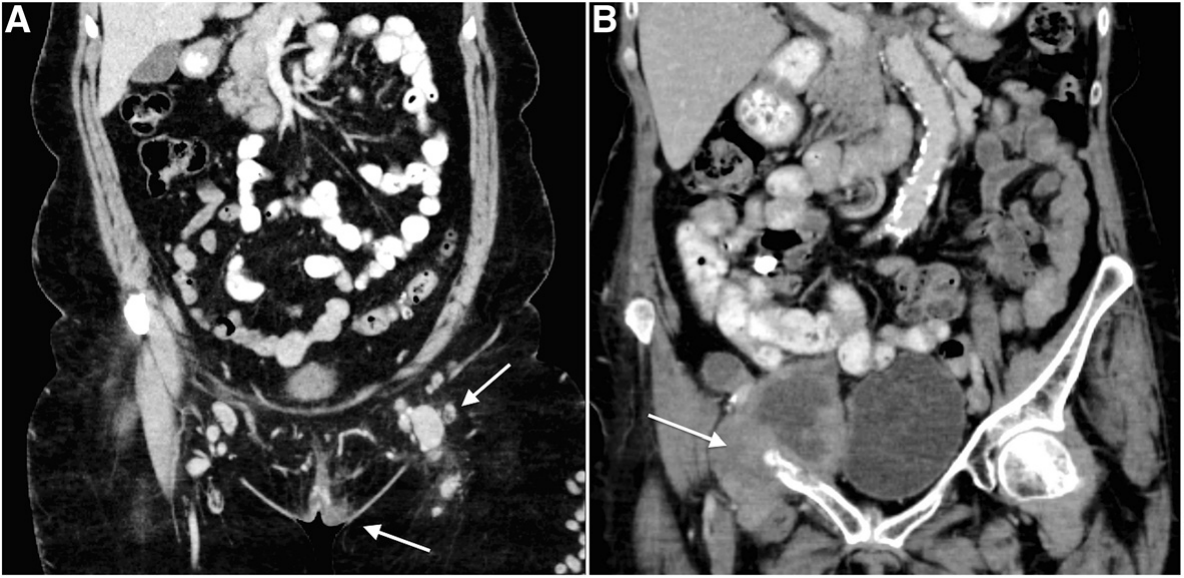
**a** A 56 year old woman with a left-sided vulvar carcinoma (*lower arrow*), palpable inguinal nodes and the corresponding CT scan showing metastases with enlargement and contrast enhancement (*upper arrow*). **b** A 74 year old woman with unexpected invasion of the ilium by lymph node metastases (*arrow*), clinically regarded resectable prior to surgery

Due to clinical findings, the treatment plan was changed in two cases (Table 4), indicating supplementary imaging (PET/CT, MRI) (Fig. 1a and b). The screening CT scan did not itself influence the treatment strategy in the study. The incidental abnormalities visualized by the CT scan were classified according to their potential clinical significance as of major, moderate or minor importance (Table 1). Two cases of incidental synchronous malignancies were found: one woman (age 61 years) with a cecal tumor with pulmonary metastases and one woman (age 56 years) with a renal tumor. In three cases, suspected pathological findings later proved to be benign. Finally, CT did not cause delay of surgery for any of the long distance, out-hospital patients. However, planning and logistics was troublesome and CT scan was omitted in this prospective study in three patients, due to missing creatinine values prior to the scan and insufficient coordination between the two departments involved in the study, and thus these patients were excluded from the study.

**Table 4:** Impact of preoperative examinations and CT scans on surgical strategy

Impact of preoperative examinations on surgical strategy			
Strategy	Pre-CT treatment plan, *n*	Post-CT treatment plan, *n*	CT changing treatment plan, *n*
Vulvectomy and unilateral SNL^a^ dissection	7	7	No change^b^
Vulvectomy and bilateral SNL dissection	13	13	No change^c^
Vulvectomy and bilateral lymphadenectomy	1	1	No change^d^
Surgery and radiotherapy or primary radiotherapy	6	4	2 patients had a changed surgical plan–x-ray and clinical findings indicated further imaging.^e^

^a^Sentinel node (SNL)

^b^Bilateral SNL dissection

^c^Unilateral lymphadenectomy

^d^Vulvectomy and unilateral lymphadenectomy

^e^Primary radiotherapy, due to invasion of the ilium (patient one). Vulvectomy and radiotherapy, due to pulmonary metastases from an adenocarcinoma of the caecum (patient two)

## Discussion

Preoperative CT scan of patients with localized vulvar carcinoma did not change the initial treatment strategy for any of the patients, nor did the high proportion of incidental findings on CT, including two cases of confirmed synchronous cancer. However, in some cases CT confirmed regional tumor spread found at the gynecological examination. This is in accordance with a retrospective study, which could not recommend a CT scan in the preoperative examination of vulvar carcinoma, due to low sensitivity and specificity in the diagnosis of lymph node metastases [11]. Preoperative palpation of the groin [15] and other imaging modalities, such as PET/CT [8, 9] and MRI [10], have also shown to be inaccurate in the detection of inguinal lymph node metastases. Currently, the only technique that appear to be effective in the detection of lymph node metastases, is either the sentinel node procedure or inguinal femoral lymphadenectomy [16, 17]. The incidental finding on the CT scanning of a synchronous renal tumor and a cecal tumor with pulmonary metastases, contributed to earlier intervention and treatment of these malignancies. Few studies exist of the frequency and clinical significance of incidental findings on CT scans. A systematic review reported the frequency of incidental findings on CT scans to be 31.1 % [18], whereas another systematic review reported clinically important findings in 7–12 % of cases [19]. In a recent retrospective study of incidental findings on CT scans of patients with prostate cancer, 20.6 % of all findings were potentially of clinical significance. Synchronous malignancy was detected in 5.9 % of patients, of which renal cancer was the most common (2.0 %) and colon cancer was found in 0.8 % of patients [20]. Taking into account the relatively small sample size and the population characteristics in the current study, the prevalence of all incidental findings (*n* = 26), renal cancer (*n* = 1) and colon cancer (*n* = 1) is comparable to that of similar studies. The benefits of managing incidental findings on CT have to be balanced to the cost, time consumption and potentially harmful consequences of further tests and treatments. At present, no studies recommend primary screening with a CT scan in any population group.

The median time intervals from the initial examination to imaging and surgical treatment were within the accepted range according to Danish guidelines. Though not quantified, due to limited CT scan capacity, the investigators spend a considerable amount of time planning and executing the CT scans, as well as adjusting for unforeseen events.

The study is limited by the relatively low number of patients included. This is primarily due to the low incidence of the disease in Denmark and the fact that only approximately half of all the Danish patients are referred to our institution. The cost of routinely adding a preoperative CT scan is not feasible, especially as the benefit of the rather time consuming procedure was low. However, any patient with surgically proven inguinal lymph node metastasis should have further imaging in order to plan the postoperative oncological treatment.

## Conclusions

If clinically feasible, surgery is mandatory in the treatment of vulvar cancer. Chest x-ray and clinical evaluation supplemented with sentinel node examination for local lymph node involvement is generally sufficient in patients without signs of advanced disease. This prospective study of evaluating a preoperative supplementary screening CT scan of the chest, abdomen and pelvis showed no clinical impact in patients without clinical indications for further imaging procedure added to the existing strategy. It did not detect unsuspected dissemination, it was inaccurate in the evaluation of lymph nodes, and it might delay the final surgery. In cases with advanced disease or when clinically indicated by pathological proven spread to the inguinal lymph nodes, CT scan–as other imaging techniques–can offer valuable information.

## Consent

All patients gave their oral and written informed consent for participating in the study, publication of the results, as well as for the given treatment.

## Abbreviations

GROINSS-V II: Groningen International Study on Sentinel Nodes in Vulvar Cancer II
UL: Ultrasound
CT: Computerized tomography
PET: Positron emission tomography
MRI: Magnetic resonance imaging
FIGO: International Federation of Gynecology and Obstetrics
IT: Ingrid Thranov
VZ: Vibeke Zobbe
MDCT: Multidetector CT
KA: Kjeld Andersen
KDP: Karen Damgaard Pedersen
